# Annotations

**Published:** 1904-07-23

**Authors:** 


### ANNOTATIONS.

### Carbolic Acid and Plague.

THE annual report of the medical officers in charge of the hospitals of the Colony of Hong Kong is particularly interesting, in at least two respects. In the first place the success attending the use of carbolic acid in the treatment of plague is demonstrated. During the last half of the outbreak the rate of mortality has been greatly diminished, and, although in the natural order of events, some fall was anticipated, because the epidemic is always more virulent at the outset, a reduction from 85 to 36 per cent. may fairly be attributed to unusual measures. It has been found that the doses of carbolic acid which a plague patient is able to take are enormous. The treatment begins with an initial dose of 144 grains in 24 hours, namely 12 grains every two hours. One European patient consumed over 2,500 grains of pure carbolic acid before his blood was pronounced free from plague bacilli. Yet, in spite of such huge doses, the poisoning of plague patients by carbolic acid is practically unknown in the Colony. The remedy was tried in Hong Kong in 1901, but with no marked result, and it is now the opinion of the medical officers that the drug administered in large doses is the most hopeful method as yet discovered of treating plague. Very wisely, however, there is no disposition to over-estimate the virtues of carbolic acid which is not, of course, a specific remedy, and the Governor of the Colony is urged in the reports to carry, if possible, into effect before next epidemic season the proposals already sanctioned for the production of a curative serum for the treatment of the malady. The other point in the report is that an improved means of diagnosis has been adopted in the Colony by which the doctors are in a position to diagnose even a very mild case. Accordingly, many persons who in former years would have been regarded as simply suffering from a minor ailment, and therefore allowed to remain at large, are under present conditions at once sent to an isolation camp. The effect of this precaution is that the danger to the community is materially lessened, and also that the patients being treated at the earliest stage of their illness, have a much better chance of making good recovery.

### An Alleged Cure for Leprosy.

A STATEMENT comes to us from Burma, which we are not at present in a position to authenticate, but which seems to be completely in harmony with the results of modern research into the nature of infectious diseases, or, at least, the infectiveness being in this case doubtful, of diseases which are liable to be prevalent in communities. It is said that Dr. Rost, the resident medical officer to the Hospital at Rangoon, has succeeded in obtaining a serum which is fatal to the bacillus of leprosy, and that patients into whom this serum has been injected have speedily shown great improvement in their condition, especially by the healing of leprous ulcers, the disappearance of tubercles, and the return of sensation to anæsthetic patches. Dr. Rost is at least well situate for rendering his work conclusive, inasmuch as leprosy is abundant in some parts of Burma, and his official position would secure to him abundant facilities for observing and treating it, and for giving publicity to any results which his observations may establish. Dr. Rost is said to have based his work upon that of Professor Koch in relation to the bacilli of tubercle, and his new serum is said to bear some relation to tuberculin. It is further alleged that his results cast doubt upon the validity of Mr. Jonathan Hutchinson's well-known hypothesis; for that, in order to obtain the required bactericide, it is necessary to remove every particle of salt from the substances from which it is prepared, or from the fluids in which the bacilli entering into its composition are cultivated. Certainly, if it be true, as alleged, that Dr. Rost prepares his serum from the leprosy bacilli, and that he is unable to keep them alive and to cultivate them if any particle of salt remain in their environment, the negative evidence will assume a somewhat more important character; but, on this hypothesis, we cannot quite understand how the bacilli live in the human body, in which salt is always present and is constantly renewed. But *qui vivra verra*, and it is obviously useless to speculate concerning a question the leading facts of which are unknown to us, and have apparently only filtered out, much as Professor Koch's hopes about tuberculin did, before the person most concerned was in a position to make any definite announcement. We can only say that the version which has reached us, if not quite intelligible in all its aspects, is still more or less in harmony with reasonable expectations, and that we shall not welcome the discovery the less warmly, if and when it is established, for having now been compelled to call attention to the elements of obscurity by which it is surrounded. We hope that Dr. Rost will soon be in a position to submit the facts of the case to the tribunal of instructed professional opinion.

### Colonisation by the Young.

COLONISTS, either at home or over sea, should be young, and considering the class from which they spring, it is almost essential that they should be removed from old associations and from kinsfolk who would drag them down. The man who has spent the best part of his life in a city slum cannot learn to endure the comparative loneliness of country, not to speak of colonial, life, and wherever it is possible he returns to his old surroundings. When he cannot he complains loudly of having been misled and deceived. Dr. Barnardo has sometimes been blamed for sending so many children to Canada who, if judged merely by their success there, had in them the making of good citizens. But they would not have made good citizens at home, for they would not have been able to shake off old associations and bad companions. Nevertheless, it is a pity to send good material abroad if it can be made use of at home, and therefore we welcome the news that the guardians of Kensington have arranged with those of Warminster to send boys from town to country. Several farmers are anxious to have the boys, and promise them good homes and decent wages. It will be for the Kensington guardians to see that this promise is kept

